# Comparison of Pediatric and Adult Glucagon-Like Peptide-1 Receptor Agonist Exposures Reported To United States Poison Centers, 2017–2024

**DOI:** 10.1007/s13181-026-01128-6

**Published:** 2026-03-05

**Authors:** Anjali Senthilkumar, Hannah L. Hays, Sandhya Kistamgari, Natalie I. Rine, Allison L. Rhodes, Christopher E. Gaw, Gary A. Smith

**Affiliations:** 1 Center for Injury Research and Policy, The Abigail Wexner Research Institute at Nationwide Children’s Hospital, 700 Children’s Drive, Columbus, OH 43205 USA; 2 University of Louisville School of Medicine, Louisville, KY USA; 3 Department of Pediatrics, The Ohio State University College of Medicine, Columbus, OH USA; 4 Central Ohio Poison Center, Nationwide Children’s Hospital, Columbus, OH USA; 5 Division of General Internal Medicine, The Ohio State University College of Medicine, Columbus, OH USA; 6 Child Injury Prevention Alliance, Columbus, OH USA

**Keywords:** Children, Semaglutide, Tirzepatide, Liraglutide, Toxicity

## Abstract

**Introduction:**

As glucagon-like peptide-1 receptor agonist (GLP-1) use has increased among children, a better understanding of the related adverse effects in this population is needed.

**Methods:**

National Poison Data System data from 2017 to 2024 were analyzed to compare characteristics and trends of exposures involving GLP-1s reported to United States (US) poison centers (PCs) among children 6–17 years old with those of adults.

**Results:**

There were 13,924 single-substance GLP-1 exposures reported to US PCs from 2017 to 2024. The rate of exposures per one million US population increased by 1,830.8% from 0.97 in 2017 to 18.79 in 2024, including a 4,805.0% increase among children 6–17 years old from 0.04 in 2017 to 1.97 in 2024, with the majority of the increase occurring after 2021. Most exposures (91.7%) were associated with no or mild effects, while moderate effects were observed in 8.0% and major effects occurred in 42 exposures; there were two deaths. Children 6–17 years old were more likely (RR: 2.66, 95% CI: 1.73–4.11) to be admitted than adults, and children 12–17 years old were more likely (RR: 1.68, 95% CI: 1.08–2.63) to experience a more serious medical outcome than adults. Children 6–17 years old with at least one clinical effect experienced vomiting (88.2%) more commonly than adults (61.3%) (RR: 1.44, 95% CI: 1.34–1.55). Additionally, exposures among children 6–17 years old were more likely to be attributable to intentional misuse (RR: 8.12, 95% CI: 6.47–10.17) than among adults.

**Conclusions:**

This study provides national-level, real-world findings that may help inform clinical practice.

**Supplementary Information:**

The online version contains supplementary material available at 10.1007/s13181-026-01128-6.

## Introduction

The global prevalence of obesity among children and adolescents is rising and is expected to increase from 10% to 20% among boys and from 8% to 18% among girls 5–19 years old between 2020 and 2035 [1]. Trends for adults aged 20 years and older are similar, with global obesity prevalence expected to increase from 14% to 23% among adult men and from 18% to 27% among adult women over this same time period [1]. In the United States (US), the prevalence of obesity among 2–19-year-old youths increased from 5% in 1963–1964 to 19% in 2017–2018 [2, 3]. This rise in obesity among youth has been associated with a marked increase in type 2 diabetes (T2DM) among children and adolescents, particularly among ethnic minority and lower socioeconomic populations [4–7]. Obesity is also a risk factor for metabolic disorders such as metabolic associated steatotic liver disease, chronic kidney disease, and cardiovascular disease [3, 8].

The increasing burden of obesity among children prompted the American Academy of Pediatrics to issue a 2023 Clinical Practice Guideline recommending that glucagon-like-peptide-1 receptor agonists (GLP-1s) be included as an adjunct to intensive lifestyle interventions to manage obesity among children 12 years and older [3]. Liraglutide (Saxenda™) and semaglutide (Wegovy™) were approved by the US Food and Drug Administration (FDA) in 2020 and 2022, respectively, to be used in combination with lifestyle modification for chronic weight management among children 12 years and older with a body mass index (BMI) equal to or *greater than the* 95^th^ percentile [9–14]. In addition, the FDA approved the use of exenatide (Bydureon™) in 2021 and dulaglutide (Trulicity™) in 2022 for use among children 10 years and older with T2DM, but not for obesity alone [15–19]. Tirzepatide is a dual action GLP-1 and glucose-dependent insulinotropic polypeptide (GIP) approved for use in adults with obesity but not yet for the pediatric population [20]. However, one tirzepatide clinical trial (SURPASS-PEDS) among participants 10–17 years old demonstrated significant improvements in glycemic control and reduction in BMI compared with placebo [21] and another trial is ongoing [22]. GLP-1 trials are also being extended to younger ages, including randomized controlled trials of liraglutide among children 6–11 years old [23, 24]. Although dispensing of GLP-1s increased by 552.4% among children 12–17 years old from 2020 to 2023, only 0.5% of children in this age group were prescribed an obesity medication in 2023 [25, 26].

Initial approval of GLP-1s by the FDA was for use in the management of T2DM among adults, including exenatide in 2005, liraglutide in 2010, dulaglutide in 2014, semaglutide in 2017, and tirzepatide in 2022 [12, 13, 17, 19, 20]. GLP-1s promote insulin secretion, suppress glucagon, delay gastric emptying, and modulate central mechanisms of satiety and reward. GIP receptor agonists enhance insulin signaling and influence adipose tissue metabolism and energy balance, contributing to enhanced weight reduction when combined with GLP-1s [4, 27–30]. The most commonly reported adverse effects of GLP-1s are gastrointestinal, including nausea, vomiting, and abdominal pain, and are minor in severity [31]. More serious effects, such as delayed gastric emptying with aspiration risk, pancreatitis and biliary complications, acute kidney or liver injury, and hypoglycemia have been reported less frequently [32–34]. However, clinical trials evaluating the efficacy of GLP-1s were not designed to capture adverse events and instead represent the experience of controlled study populations [33]. Real-world data are limited, especially for the pediatric population, and a comprehensive comparison of GLP-1-related adverse effects between the pediatric and adult populations is needed as the use of these medications expands among children. It should not be assumed that adverse effects among adults will necessarily be the same among children and adolescents.

The objective of this study is to characterize exposures involving GLP-1s and dual GIP/GLP-1s reported to US poison centers (PCs) among children 6–17 years old from 2017 to 2024, and to compare exposure characteristics and trends with those reported among adults 18 years and older. These findings build on our previous research [35] about GLP-1 exposures reported to US PCs, which did not focus on comparing the pediatric and adult age groups and included all reasons for exposure (without specifically identifying adverse drug reactions) and individuals of all ages (including children < 6 years old). This study aims to complement clinical trial data by providing national population-level, real-world data to help inform clinical practice.

## Methods

### Data Sources

We analyzed data from the National Poison Data System (NPDS), which is the data warehouse for the regional PCs in the US and its territories [36, 37]. At each PC, Specialists in Poison Information record data from calls into the PC’s electronic medical record using established codes and protocols. Data from all PC calls are then automatically uploaded to the NPDS database in near real-time [38]. Intercensal and postcensal population estimates (including age group-specific and sex-specific estimates) from the US Census Bureau were used to calculate population-specific rates of exposure [39, 40]. This study was determined to be exempt by the institutional review board of the authors’ institution.

### Case Selection Criteria

This study included single-substance exposures involving only reported GLP-1 exposure among individuals 6 years and older reported to US PCs from January 1, 2017, to December 31, 2024. Exposures involving GLP-1s were identified using NPDS product codes. Because we sought to capture exposures that occur during use of these medications for therapeutic purposes, we limited cases to those with a reason for exposure identified as unintentional-therapeutic error, adverse drug reaction, or intentional-misuse. The NPDS defines intentional-misuse as “an exposure resulting from the intentional improper or incorrect use of a substance for reasons other than the pursuit of a psychotropic effect” [38]. Cases were excluded if the individual’s age was unknown (specifically, including “unknown ≤ 19 years,” “unknown ≥ 20 years,” “20s,” and “60s”) or the medical outcome was coded as “confirmed non-exposure” or “unrelated exposure.”

### Study Variables

Study variables included age, sex, year of exposure, reason for exposure, exposure site, product involved, related clinical effects, therapies performed, highest level of health care received, and medical outcome. We grouped age into 6–11 years (younger children), 12–17 years (older children), 18–25 years (young adults), 26–65 years (mid-age adults), and > 65 years (older adults). For some analyses, the pediatric age groups 6–11 and 12–17 years were combined and the adult age groups 18–25, 26–65, and > 65 years were combined. Exposure site was grouped into residence, other, and unknown. GLP-1s were categorized using NPDS product codes as semaglutide, liraglutide, tirzepatide, and other GLP-1s (including albiglutide, dulaglutide, exenatide, lixisenatide, and compounded GLP-1s). These NPDS product codes were initially established prior to 2017, with the exception of semaglutide (2018), tirzepatide (2022), and compounded GLP-1s (2024). Definitions of related clinical effects are found in the NPDS Coding Users’ Manual [38].

Highest level of health care received is categorized by the NPDS as (1) no treatment received at a healthcare facility, (2) treated/evaluated and released, (3) admitted to a critical care unit, (4) admitted to a non-critical care unit, (5) admitted to a psychiatric facility, (6) refused referral/did not arrive at a healthcare facility, and (7) unknown (includes patient lost to follow up or left against medical advice) [38]. Admission to a critical care unit and admission to a non-critical care unit were combined as “medical admissions” during analyses.

The NPDS categorizes medical outcome as (1) no effect, (2) minor effect (minimal symptoms that generally resolve rapidly), (3) moderate effect (more pronounced, prolonged, or systemic symptoms than minor effect), (4) major effect (symptoms are life-threatening or result in significant disability or disfigurement), (5) death, (6) not followed (includes judged as a non-toxic exposure or minimal clinical effects possible), and (7) unknown (unable to follow, judged as a potentially toxic exposure) [38]. Moderate effect, major effect, and death were combined as “more serious medical outcomes” during analyses.

### Statistical Analysis

We used IBM SPSS Statistics 29.0 (IBM Corp, Armonk, NY) and SAS 9.4 (SAS Institute, Inc, Cary, NC) for analyses. Descriptive statistics and population-based exposure rates were calculated. Piecewise and simple linear regression models were used, as appropriate, to determine the statistical significance of trends by evaluating whether the null hypothesis of slope = 0 could be rejected using Student’s t-test; regression modeling was not performed. The level of statistical significance was set at α = 0.05. Consistent with our study’s retrospective cohort design, we calculated risk ratios (RRs) with corresponding 95% confidence intervals (CIs) to assess the magnitude of associations between characteristics (such as age group, sex, or product category) and outcomes of interest (such as medical outcome or medical admission).

## Results

### General Characteristics

There were 13,924 single-substance exposures involving GLP-1s among individuals 6 years and older reported to US PCs from 2017 to 2024 that met study inclusion criteria. More than three-fourths (77.3%) were female and 98.9% of exposure incidents occurred at a residence (Table 1). The pediatric age group accounted for 1.3% (*n* = 178) of exposures, including 0.4% (*n* = 49) among children 6–11 years old and 0.9% (*n* = 129) among those 12–17 years old. Overall, most exposures involved semaglutide (62.3%), followed by tirzepatide (12.8%) and liraglutide (10.7%).

**Table 1:** Characteristics of GLP-1 Exposures Reported to United States Poison Centers by Age Group, NPDS 2017–2024

					Age Groups			
**Characteristics**			6–11 Years	12–17 Years	18–25 Years	26–65 Years	> 65 Years	Total
			n (%)^a^	n (%)^a^	n (%)^a^	n (%)^a^	n (%)^a^	n (%)^a^
**Sex**								
Male			23 (46.9)	39 (30.2)	79 (14.3)	2,211 (20.9)	803 (30.8)	3,155 (22.7)
Female			26 (53.1)	90 (69.8)	475 (85.7)	8,362 (79.1)	1,807 (69.2)	10,760 (77.3)
Unknown			0	0	2	7	0	9
**Exposure site**								
Residence			48 (98.0)	127 (99.2)	548 (98.9)	10,446 (98.9)	2,585 (99.1)	13,754 (98.9)
Other location			1 (2.0)	1 (0.8)	6 (1.1)	116 (1.1)	24 (0.9)	148 (1.1)
Unknown			0	1	2	18	1	22
**Reason for exposure**								
Therapeutic error			38 (77.6)	75 (58.1)	394 (70.9)	9,065 (85.7)	2,431 (93.1)	12,003 (86.2)
Intentional misuse			10 (20.4)	48 (37.2)	76 (13.7)	439 (4.1)	37 (1.4)	610 (4.4)
Adverse drug reaction			1 (2.0)	6 (4.7)	86 (15.5)	1,076 (10.2)	142 (5.4)	1,311 (9.4)
**Highest level of health care received**								
No healthcare facility treatment received			20 (45.5)	49 (41.9)	284 (57.4)	6,857 (69.4)	1,874 (75.1)	9,084 (69.7)
Treated/evaluated and released			18 (40.9)	52 (44.4)	161 (32.5)	2,321 (23.5)	462 (18.5)	3,014 (23.1)
Medical admission			5 (11.4)	14 (12.0)	20 (4.0)	412 (4.2)	119 (4.8)	570 (4.4)
Admitted to critical care unit			1 (2.3)	5 (4.3)	1 (0.2)	65 (0.7)	20 (0.8)	92 (0.7)
Admitted to non-critical care unit			4 (9.1)	9 (7.7)	19 (3.8)	347 (3.5)	99 (4.0)	478 (3.7)
Admitted to psychiatric facility			0 (0.0)	0 (0.0)	1 (0.2)	2 (0.0)	0 (0.0)	3 (0.0)
Refused referral/did not arrive at healthcare facility			1 (2.3)	2 (1.7)	29 (5.9)	287 (2.9)	42 (1.7)	361 (2.8)
Unknown^b^			5	12	61	701	113	892
**Medical outcome**								
No effect			16 (35.6)	10 (8.3)	28 (5.6)	1,165 (11.7)	480 (19.2)	1,699 (12.9)
Minor effect			6 (13.3)	49 (40.5)	165 (32.9)	2,508 (25.1)	428 (17.1)	3,156 (24.0)
More serious medical outcome			2 (4.4)	17 (14.0)	52 (10.4)	870 (8.7)	156 (6.2)	1,097 (8.3)
Moderate effect			2 (4.4)	16 (13.2)	50 (10.0)	838 (8.4)	147 (5.9)	1,053 (8.0)
Major effect			0 (0.0)	1 (0.8)	2 (0.4)	31 (0.3)	8 (0.3)	42 (0.3)
Death			0 (0.0)	0 (0.0)	0 (0.0)	1 (0.0)	1 (0.0)	2 (0.0)
Not followed^c^			21 (46.7)	45 (37.2)	257 (51.2)	5,436 (54.5)	1,442 (57.5)	7,201 (54.7)
Unknown^d^			4	8	54	601	104	771
**Product category**								
Semaglutide			26 (53.1)	91 (70.5)	404 (72.7)	6,688 (63.2)	1,459 (55.9)	8,668 (62.3)
Liraglutide			7 (14.3)	12 (9.3)	39 (7.0)	1,103 (10.4)	327 (12.5)	1,488 (10.7)
Tirzepatide			2 (4.1)	14 (10.9)	75 (13.5)	1,442 (13.6)	249 (9.5)	1,782 (12.8)
Other^e^			14 (28.6)	12 (9.3)	38 (6.8)	1,347 (12.7)	575 (22.0)	1,986 (14.3)
**Total (Row %)**			**49 (0.4)**	**129 (0.9)**	**556 (4.0)**	**10**,**580 (76.0)**	**2**,**610 (18.7)**	**13**,**924 (100.0)**

^**a**^ Column percentages may not add to 100.0% due to rounding error

^b^ Includes: patient lost to follow-up, left against medical advice, and unknown

^c^ Includes: “not followed (minimal clinical effects possible)” and “not followed (judged as a non-toxic exposure)”

^d^ Includes: “unable to follow (potentially toxic exposure)”

^e^ Includes: albiglutide, dulaglutide, exenatide, and compounded GLP-1s

### Reason for Exposure

Most (86.2%) exposures in this study were therapeutic errors, followed by adverse drug reactions (9.4%) and intentional misuse (4.4%) (Table 1). Compared with adults 18 years and older, children 6–17 years old were less likely to experience therapeutic errors (RR: 0.73, 95% CI: 0.66–0.82) or adverse drug reactions (RR: 0.41, 95% CI: 0.20–0.86).Although intentional misuse accounted for 4.4% of exposures among all individuals in the study, it represented 32.6% of exposures among children 6–17 years old. Exposures among children 6–17 years old were more likely to be attributable to intentional misuse (RR: 8.12, 95% CI: 6.47–10.17) than among adults 18 years and older.

### Highest Level of Health Care Received

Most exposures did not receive treatment at a healthcare facility (69.7%) or were treated/evaluated and released (23.1%) (Table 1). There were 570 medical admissions (4.4%), including 92 (0.7%) admissions to a critical care unit and 478 (3.7%) to a non-critical care unit. A higher proportion of 6–11-year-old (11.4%) and 12–17-year-old (12.0%) children were admitted compared with adults 18 years and older (4.3%), and children 6–17 years old were more likely to be medically admitted (RR: 2.66, 95% CI: 1.73–4.11) than adults. Among all individuals, 4.8% of semaglutide exposures were medically admitted (Table 2) and were more likely to be medically admitted (RR: 1.25, 95% CI: 1.05–1.49) than exposures involving other types of GLP-1s combined.

### Medical Outcome

Most exposures were associated with minor effects (24.0%) or were not followed (54.7%) because the exposure was judged by PC personnel as non-toxic or only to have minimal clinical effects possible (Table 1). More serious medical outcomes were seen in 8.3% of individuals, including 8.0% (*n* = 1,053) with moderate effects, 0.3% (*n* = 42) with major effects, and 2 deaths. Children 6–17 years old experienced the highest proportion (10.7%) of moderate effects among all age groups. Although children 12–17 years old were more likely to experience a more serious medical outcome than adults 18 years and older (RR: 1.68, 95% CI: 1.08–2.63), the broader pediatric age group (6–17 years old) was not (RR: 1.36, 95% CI: 0.89–2.09). Semaglutide had the highest proportion of more serious medical outcomes (9.3%), followed by tirzepatide (8.2%) and liraglutide (5.7%) (Table 2). Semaglutide was more likely to be associated with a more serious medical outcome than other GLP-1s combined (RR: 1.35, 95% CI: 1.19–1.53).

**Table 2:** Characteristics of GLP-1 Exposures Reported to United States Poison Centers by Product Category, NPDS 2017–2024

		Product Categories			
**Characteristics**	Semaglutide	Liraglutide	Tirzepatide	Other^a^	Total
	n (%)^b^	n (%)^b^	n (%)^b^	n (%)^b^	n (%)^b^
**Sex**					
Male	1,937 (22.4)	326 (21.9)	323 (18.1)	569 (28.7)	3,155 (22.7)
Female	6,723 (77.6)	1,161 (78.1)	1,459 (81.9)	1,417 (71.3)	10,760 (77.3)
Unknown	8	1	0	0	9
**Age group**					
6–11 years	26 (0.3)	7 (0.5)	2 (0.1)	14 (0.7)	49 (0.4)
12–17 years	91 (1.1)	12 (0.8)	14 (0.8)	12 (0.6)	129 (0.9)
18–25 years	404 (4.7)	39 (2.6)	75 (4.2)	38 (1.9)	556 (4.0)
26–65 years	6,688 (77.2)	1,103 (74.1)	1,442 (80.9)	1,347 (67.8)	10,580 (76.0)
>65 years	1,459 (16.8)	327 (22.0)	249 (14.0)	575 (29.0)	2,610 (18.7)
**Exposure site**					
Residence	8,576 (99.1)	1,470 (99.0)	1,762 (99.0)	1,946 (98.2)	13,754 (98.9)
Other location	79 (0.9)	15 (1.0)	18 (1.0)	36 (1.8)	148 (1.1)
Unknown	13	3	2	4	22
**Reason for exposure**					
Therapeutic error	7,323 (84.5)	1,382 (92.9)	1,510 (84.7)	1,788 (90.0)	12,003 (86.2)
Intentional misuse	429 (4.9)	33 (2.2)	84 (4.7)	64 (3.2)	610 (4.4)
Adverse drug reaction	916 (10.6)	73 (4.9)	188 (10.5)	134 (6.7)	1,311 (9.4)
**Highest level of health care received**					
No healthcare facility treatment received	5,101 (63.5)	1,123 (78.8)	1,358 (80.6)	1,502 (79.3)	9,084 (69.7)
Treated/evaluated and released	2,277 (28.4)	221 (15.5)	235 (13.9)	281 (14.8)	3,014 (23.1)
Medical admission	384 (4.8)	55 (3.8)	51 (3.1)	80 (4.2)	570 (4.4)
Admitted to critical care unit	55 (0.7)	9 (0.6)	13 (0.8)	15 (0.8)	92 (0.7)
Admitted to non-critical care unit	329 (4.1)	46 (3.2)	38 (2.3)	65 (3.4)	478 (3.7)
Admitted to psychiatric facility	3 (0.0)	0 (0.0)	0 (0.0)	0 (0.0)	3 (0.0)
Refused referral/did not arrive at healthcare facility	262 (3.3)	26 (1.8)	41 (2.4)	32 (1.7)	361 (2.8)
Unknown^c^	641	63	97	91	892
**Medical outcome**					
No effect	861 (10.6)	275 (19.2)	170 (10.0)	393 (20.5)	1,699 (12.9)
Minor effect	2,305 (28.4)	230 (16.1)	302 (17.8)	319 (16.6)	3,156 (24.0)
More serious medical outcome	757 (9.3)	82 (5.7)	139 (8.2)	119 (6.2)	1,097 (8.3)
Moderate effect	729 (9.0)	78 (5.4)	131 (7.7)	115 (6.0)	1,053 (8.0)
Major effect	27 (0.3)	4 (0.3)	7 (0.4)	4 (0.2)	42 (0.3)
Death	1 (0.0)	0 (0.0)	1 (0.1)	0 (0.0)	2 (0.0)
Not followed^d^	4,185 (51.6)	845 (59.0)	1,086 (64.0)	1,085 (56.6)	7,201 (54.7)
Unknown^e^	560	56	85	70	771
**Total (Row %)**	**8**,**668 (62.3)**	**1**,**488 (10.7)**	**1**,**782 (12.8)**	**1**,**986 (14.3)**	**13**,**924 (100.0)**

^a^ Includes: albiglutide, dulaglutide, exenatide, and compounded GLP-1s

^b^ Column percentages may not add to 100.0% due to rounding error

^c^ Includes: patient lost to follow-up, left against medical advice, and unknown

^d^ Includes: “not followed (minimal clinical effects possible)” and “not followed (judged as a non-toxic exposure)”

^e^ Includes: “unable to follow (potentially toxic exposure)”

The first of the two reported fatalities was a 39-year-old female, who experienced an adverse drug reaction to tirzepatide. Her clinical course was complicated by bowel ischemia and resection, suspected gastrointestinal bleeding, and repeat episodes of cardiac arrest. The relative contribution to fatality of tirzepatide was judged by the reporting PC as “contributory” and by America’s Poison Centers as “unknown;” this case was previously reported [35]. The second fatality was a 79-year-old male, who died following a therapeutic error involving semaglutide with a scenario of “medication doses given/taken too close together.” He experienced confusion, electrolyte abnormality, hypoglycemia, and asystole; and the reporting PC indicated that the clinical effects were “unknown if related.”

Among the 42 exposures associated with major effects, one occurred in the pediatric age group. This was a 13-year-old female, who experienced an adverse drug reaction to semaglutide. She was admitted to a non-critical care unit and experienced vomiting, acidosis, electrolyte abnormality, hypoglycemia, a prolonged QT interval, and tachycardia. She received intravenous fluids, benzodiazepines, and antiemetics.

### Related Clinical Effects and Therapies Performed

Among the 6,391 individuals with at least one related clinical effect recorded, the most common related clinical effects were nausea (*n* = 4,191, 65.6%), vomiting (*n* = 3,945, 61.7%), abdominal pain (*n* = 969, 15.2%), and diarrhea (*n* = 710, 11.1%); this was true across all age groups and GLP-1 product categories. Children 6–17 years old were more likely to experience at least one related clinical effect (57.3%, 102/178) than adults 18 years and older (45.8%, 6,289/13,746) (RR: 1.25, 95% CI: 1.10–1.42). Children 6–17 years old with at least one related clinical effect experienced vomiting (*n* = 90, 88.2%) more commonly than adults 18 years and older (*n* = 3,855, 61.3%) (RR: 1.44, 95% CI: 1.34–1.55).

Among the 4,974 individuals with documentation of at least one therapy performed, the most common therapies performed were dilute/irrigate/wash (*n* = 1,962, 39.4%), food/snack given (*n* = 1,916, 38.5%), antiemetics (*n* = 1,473, 29.6%), and intravenous fluids (*n* = 1,229, 24.7%). Children 6–17 years old were more likely to receive at least one therapy (47.2%, 84/178) than adults 18 years and older (35.6%, 4,890/13,746) (RR: 1.33, 95% CI: 1.13–1.55). Compared with other age groups, food/snack was given proportionally more often among 6–11-year-old children (*n* = 10, 50.0%) and antiemetics (*n* = 38, 59.4%) and intravenous fluids (*n* = 30, 46.9%) were administered proportionally more often among children 12–17 years old.

### Trends

**Fig. 1:**
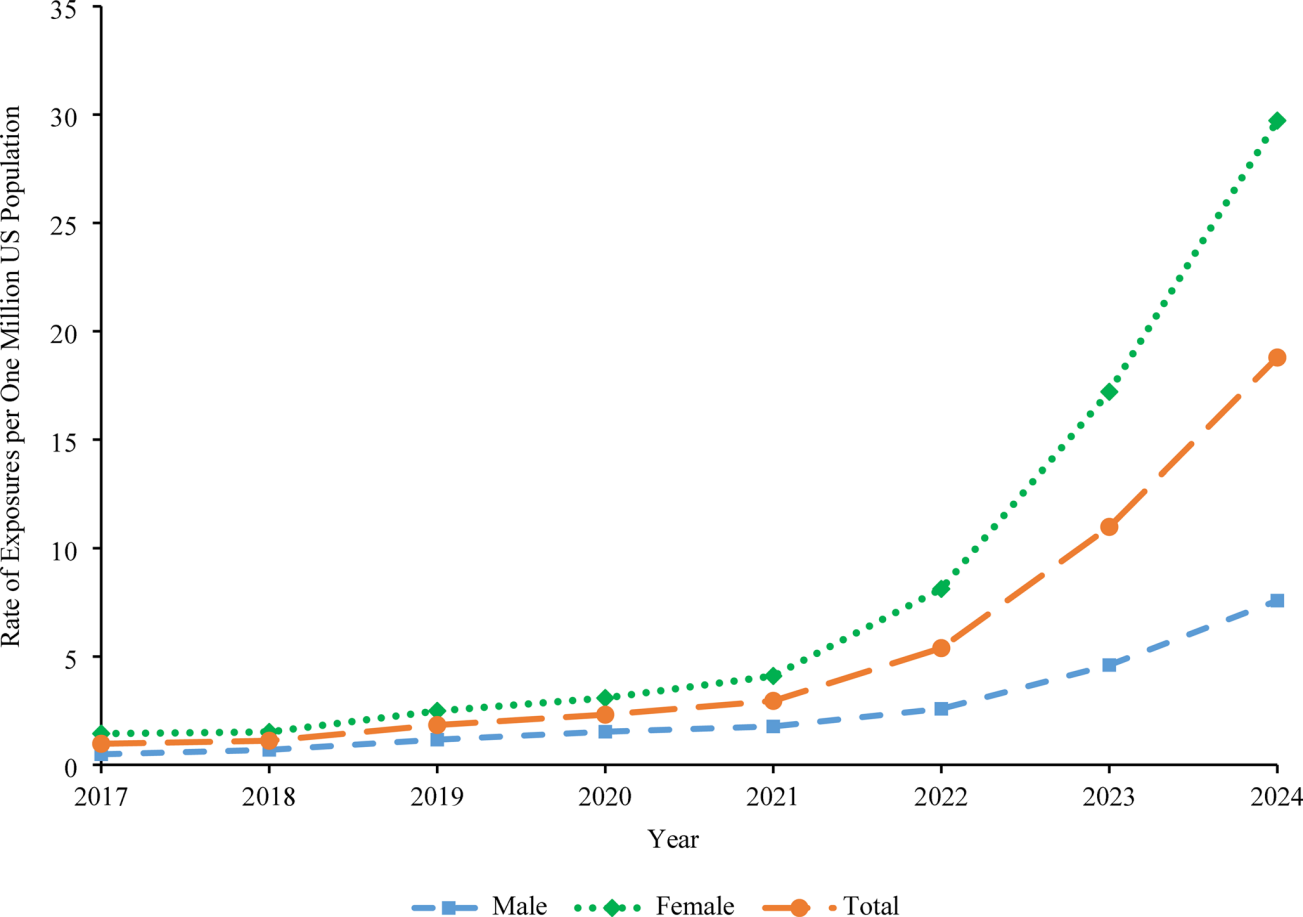
Rate of GLP-1 Exposures Reported to United States Poison Centers by Sex, NPDS 2017–2024

The rate of single-substance exposures per one million US population involving GLP-1s among individuals 6 years and older reported to US PCs increased from 0.97 in 2017 to 2.95 in 2021 (*P* = 0.601), followed by a rapid increase of 536.3% from 2021 to 18.79 in 2024 (*P* < 0.001). Overall, this represented a non-linear rate increase of 1,830.8% from 2017 to 2024 (*P* < 0.001). This trend was seen for both males and females, with the highest rate and largest increase observed among females, which increased by 624.4% from 4.10 in 2021 to 29.72 in 2024 (*P* < 0.001) (Fig. 1).

**Fig. 2:**
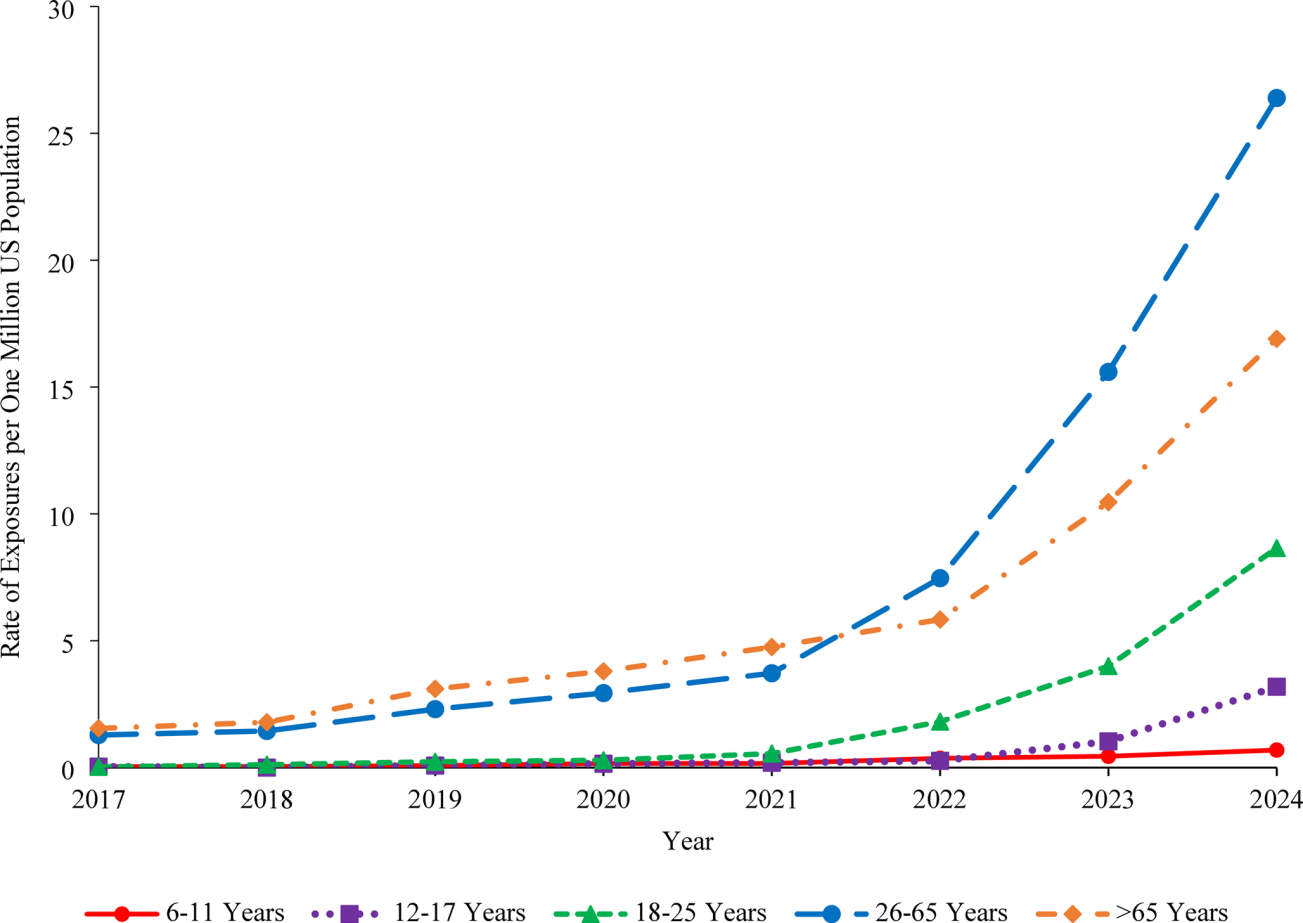
Rate of GLP-1 Exposures Reported to United States Poison Centers by Age Group, NPDS 2017–2024

Older age groups generally had higher GLP-1 exposure rates than younger age groups, with the exception that the rate for 25–65-year-old adults exceeded that for individuals > 65 years old starting in 2022 (Fig. 2). Comparing trends within age groups, the GLP-1 exposure rate increased non-linearly by 4,805.0% (*P* = 0.020) from 0.04 in 2017 to 1.97 in 2024 among children 6–17 years old compared with 1,799.2% (*P* = 0.006) among adults 18 years and older from 1.16 in 2017 to 21.98 in 2024, with more rapid rate increases observed starting in 2021. The exposure rate among children 6–17 years old increased by 1,013.1% (*P* < 0.001) from 0.18 in 2021 to 1.97 in 2024; among the 6–11-year-old sub-group, the rate increased from 0.16 in 2021 to 0.69 in 2024 (324.2%, *P* < 0.001) and among the 12–17-year-old sub-group, it increased from 0.19 in 2021 to 3.19 in 2024 (1,568.8%, *P* < 0.001).

**Fig. 3:**
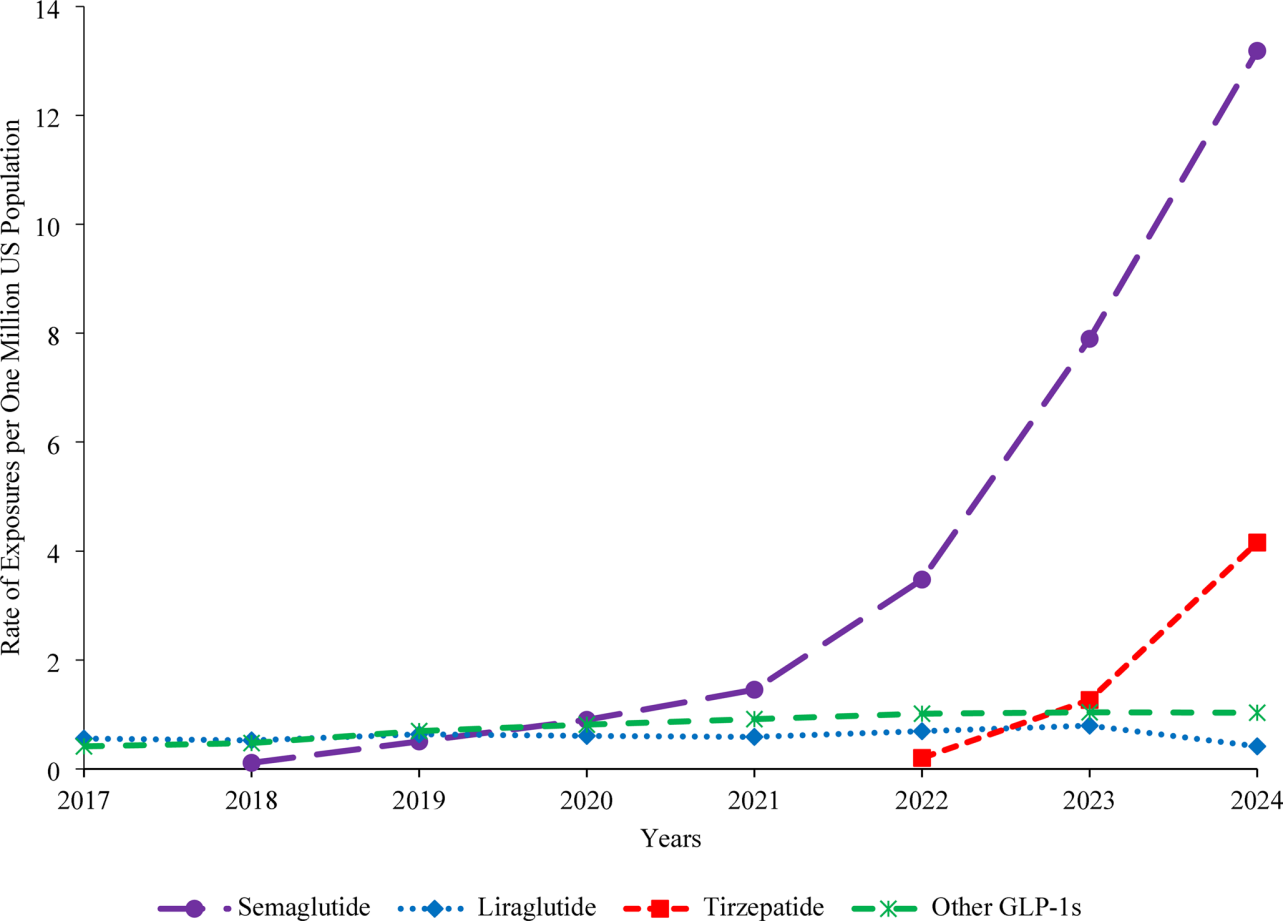
Rate of GLP-1 Exposures Reported to United States Poison Centers by Product Category, NPDS 2017–2024

After the NPDS product code for semaglutide was established in 2018, the rate of semaglutide exposures per one million US population increased slowly (1,196.6%, *P* = 0.474) from 0.11 in 2018 to 1.46 in 2021, followed by a rapid 806.0% increase from 1.46 in 2021 to 13.19 in 2024 (*P* < 0.001) (Fig. 3). The liraglutide exposure rate increased steadily by 42.6% from 0.56 in 2017 to 0.80 in 2023 (*P* = 0.018) and then decreased by 47.8% to 0.42 in 2024 (*P* = 0.005). After the NPDS product code for tirzepatide was established in 2022, the tirzepatide exposure rate increased by 1,955.3% from 0.20 in 2022 to 4.16 in 2024 (*P* < 0.001). Other GLP-1 exposures increased by 144.5% from 0.42 in 2017 to 1.01 in 2022 (*P* < 0.001) and then remained relatively unchanged through 2024 (*P* = 0.911).

The rate of more serious medical outcomes per one million US population involving GLP-1s among individuals of all ages increased by 177.5% from 0.07 in 2017 to 0.18 in 2021 (*P* = 0.934), followed by an increase of 808.6% from 2021 to 1.67 in 2024 (*P* < 0.001) (Appendix 1). This trend was similar for both sexes with higher rates observed among females. The rates increased by 574.1% (*P* = 0.001) from 0.09 in 2021 to 0.57 in 2024 among males and by 877.6% (*P* < 0.001) from 0.28 in 2021 to 2.75 in 2024 among females. The rate of more serious medical outcomes was highest among the 26–65-year-old age group throughout the study period and increased by 863.2% from 0.25 in 2021 to 2.41 in 2024 (*P* < 0.001) (Appendix 2). In addition, the rate increased by 2,783.3% from 0.03 in 2021 to 0.84 in 2024 among adults 18–25 years old (*P* < 0.001) and by 362.1% from 0.25 in 2021 to 1.16 in 2024 (*P* < 0.001) among older adults > 65 years old. An analysis of secular rate trends for the pediatric age group was not performed because of the small number of more serious medical outcomes in that group; however, an increase in the rate was noted between 2023 and 2024. The rate of more serious medical outcomes associated with semaglutide was higher than those for the other GLP-1 product categories starting in 2019 and increased rapidly by 2,455.2% from 0.05 in 2020 to 1.24 in 2024 (*P* = 0.002) (Appendix 3). The rate of more serious medical outcomes associated with liraglutide showed an insignificant increase of 89.6% from 0.02 in 2017 to 0.03 in 2024 (*P* = 0.139). In addition, the rate associated with tirzepatide increased by 899.8% from 0.03 in 2022 to 0.32 in 2024 (*P* < 0.001).

The rate of medical admissions per one million US population involving GLP-1s among individuals of all ages increased by 167.8% from 0.05 in 2017 to 0.14 in 2021 (*P* = 0.077), followed by a 369.0% increase from 2021 to 0.67 in 2024 (*P* < 0.001) (Appendix 4). The rates for both sexes increased during the study period with higher rates observed among females. The rate increased by 64.0% (*P* = 0.029) from 0.12 in 2021 to 0.20 in 2024 among males and by 600.7% (*P* < 0.001) from 0.16 in 2021 to 1.12 in 2024 among females. An analysis of secular rate trends for the pediatric age group was not performed because of the small number of medical admissions in that group; however, the rates for 6–11-year-old and 12–17-year-old children increased in 2024, with the rate of 0.38 for children 12–17 years old exceeding that for 18–25-year-old individuals in that year. Admission rates among 18–25 years old increased by 958.7% from 0.03 in 2020 to 0.31 in 2024 (*P* = 0.008) (Appendix 5). In addition, admission rates among individuals 26–65 years old increased by 889.7% from 0.09 in 2020 to 0.86 in 2024 (*P* < 0.001), and admission rates among older adults > 65 years increased by 460.9% from 0.12 in 2020 to 0.67 in 2024 (*P* < 0.001). The medical admission rate associated with semaglutide was higher than those of other GLP-1s starting in 2019 and increased rapidly by 1,722.1% from 0.03 in 2020 to 0.53 in 2024 (*P* < 0.001) (Appendix 6). The admission rate for liraglutide showed little change during the study period (*P* > 0.05) and the rate for tirzepatide increased by 527.3% from 0.02 in 2022 to 0.10 in 2024 (*P* = 0.002).

## Discussion

This national study showed an increase of 1,830.8% in the rate of GLP-1 exposures reported to US PCs from 2017 to 2024, with most of the increase occurring after 2021. This increase paralleled the escalation in dispensing of these medications for T2DM and chronic weight management. For example, among the pediatric age group from 2021 to 2024, GLP-1 exposure rates increased by 324.2% among children 6–11 years old and 1,568.8% among those 12–17 years old. This mirrored an increase of 552.4% in the dispensing of GLP-1s among children 12–17 years old from 1,653 in 2020 to 10,785 in 2023, based on reports to the IQVIA Longitudinal Prescription Database from almost 94% of US retail pharmacies [26]. Another study of claims data reported a 196.4% increase in dispensing of GLP-1s among US adults without comorbid alcohol use disorder from 2.21 prescriptions per 100 person-years in 2016 to 6.55 in 2024 [41]. These increases were likely accelerated by the expanded FDA indications and professional endorsement by the American Academy of Pediatrics supporting GLP-1 use among children 12 years and older with obesity as an adjunct to lifestyle interventions [3, 10, 14]. Although most exposures were associated with no or minor effects, the rates of both medical admissions and more serious medical outcomes increased among all age groups during the study period. Consistent with two previous studies that analyzed PC data, most exposures in our study involved females [35, 42]. Our findings are also consistent with studies that show that females 12–17 years received a higher and faster growing proportion of prescriptions for GLP-1s compared with males from 2020 to 2023 [25, 26]. Reports from US retail pharmacies during this period revealed that among adolescents 12–17 years old, prescriptions for GLP-1s increased 504% (from 692 to 4178) for males and 588% (from 961 to 6607) for females [25, 26].

A notable finding in this study was the difference in medical admissions and medical outcomes of the pediatric age group compared with adults. Children 6–17 years old were 2.7 times more likely to be medically admitted than adults 18 years and older. Many factors may influence the decision to medically admit a patient, including the type and severity of symptoms, circumstances of the exposure event, available resources in the home and access to medical follow-up care, comorbidities, healthcare provider experience and characteristics, and patient demographics, including age. In addition, the 12–17-year age group was 1.7 times more likely to experience more serious medical outcomes than adults in our study, and although most pediatric admissions were to non-critical care units, this pattern may suggest that children experience more severe or prolonged gastrointestinal effects, dehydration, or other clinically significant effects before seeking medical evaluation. In addition, because pediatric clinicians are often less familiar with this medication class, symptoms may be mistakenly attributed to gastroenteritis, leading to delayed diagnosis and prolonged symptoms, which highlights the need for a high index of suspicion in pediatric patients. Anticipatory guidance about prevention and early recognition of adverse effects should be a priority during clinical management of pediatric patients receiving GLP-1s.

Unintentional therapeutic error was the most common reason for exposure across all age groups, consistent with prior PC studies among individuals older than 6 years [35, 42]. However, a key difference between the pediatric and adult age groups in this study was the proportion of exposures attributable to intentional misuse. Intentional misuse accounted for approximately one-third of exposures among children 6–17 years old, and exposures in this age group were more than eight times more likely to be attributable to intentional misuse than among adults. The increase in intentional misuse among the pediatric age group (especially among adolescents) may represent the influence of several factors, such as heightened body image concerns, increased visibility of GLP-1s for weight loss through social media, and the relative ease of unsupervised self-administration of these injectable medications [43, 44]. Given that childhood obesity strongly predicts adolescent obesity, adolescents prescribed GLP-1s for obesity often have longstanding weight-related distress [45, 46]. FDA-approved use among adolescents is limited to those who require an additional treatment option to manage their obesity [3], suggesting recipients have struggled with weight loss for years. Consequently, overuse or unsupervised dose escalation may represent an attempt to accelerate weight loss [43]. Health care providers should incorporate routine screening for misuse behaviors among patients using GLP-1s, and PC staff should remain alert to calls that may represent intentional misuse, particularly among adolescents.

Consistent with prior clinical trial data, the most common related clinical effects in our study were gastrointestinal, including nausea, vomiting, abdominal pain, and diarrhea [34, 47–49]. Dehydration and acute kidney injury have been previously reported among patients with more severe gastrointestinal effects [50, 51]. Additionally, in our study, vomiting was more common among the pediatric age group, especially those 12–17 years old, than adults. Among pediatric exposures, food/snack administration, antiemetics, and intravenous fluids were frequent therapeutic interventions.

Semaglutide accounted for more than 60% of all exposures and was 1.25 times more likely to be associated with medical admission and 1.35 times more likely to be associated with a more serious medical outcome than other GLP-1s. The limitations of available data in the NPDS precluded our ability to determine the reasons for this greater relative severity of semaglutide-associated exposures.

Given the rapid increase in GLP-1 exposures reported to US PCs, patient and caregiver education about prevention of therapeutic errors, possible adverse clinical effects, and when to seek medical advice or attention is essential. For the pediatric population, counseling should also include discussions about mental health, body image, parameters for healthy weight loss, optimal nutrition and hydration, and risks of intentional misuse.

### Study Limitations

This study has several limitations. PCs do not receive reports of all GLP-1 adverse events; therefore, this study underestimates the true number of these exposures. The NPDS includes voluntary, self-reports of exposures, and PCs and America’s Poison Centers cannot completely verify the accuracy of this information. Exposure calls to PCs do not necessarily represent a true poisoning or overdose. NPDS data are also limited by possible reporting bias, misclassification, and incomplete follow-up. The lack of prescribing denominator data and information about comorbidities, race/ethnicity, socioeconomic status, and the indication for prescribing the GLP-1 for an individual (such as obesity or T2DM) were also limitations. The medication dose and route of exposure were not considered during analyses and only single-substance exposures were included in this study. Length of hospital stay for admitted patients is not available in the NPDS and therefore was not included in analyses. Although frequencies and rates for the age groups 6–11 years and 12–17 years are shown separately in the tables, figures, and appendices, analyses were also performed on the combined 6–17-year-old age group. Several product codes were established during the study time period (semaglutide in 2018, tirzepatide in 2022, and compounded GLP-1 products in 2024), which may have influenced observed trends. In addition, the recent addition of the product code for compounded GLP-1 products precluded analysis of this category. The rarity of pediatric exposures during some years also limited analysis of trends. Despite these limitations, the NPDS is a comprehensive, standardized national database commonly used for epidemiologic investigations of medication exposures.

## Conclusions

This study builds on our previous research [35] by comparing GLP-1 exposure characteristics and trends among children with those of adults. Our findings provide real-world data at the national population level that may help inform clinical practice. They revealed differences between children and adults, including that children 6–17 years old were more likely than adults to have exposures attributable to intentional misuse, be admitted, experience a more serious medical outcome (children 12–17 years old only), or experience vomiting. The findings reinforce the need for ongoing pharmaco-surveillance, particularly as pediatric use of GLP-1s expands.

## Supplementary Information

Below is the link to the electronic supplementary material.

Supplementary Material 1 (DOCX 46.0 KB)

## Data Availability

Data analyzed in this study were from the National Poison Data System, which is owned and managed by America’s Poison Centers. Data requests should be submitted to America’s Poison Centers.

## Abbreviations

BMI: body mass index
CI: confidence interval
FDA: United States Food and Drug Administration
GIP: glucose-dependent insulinotropic polypeptide
GLP-1: glucagon-like peptide-1 receptor agonist
NPDS: National Poison Data System
PC: poison center
RR: risk ratio
T2DM: type 2 diabetes mellitus
US: United States
