# Supplementary material for: Comparison of Pediatric and Adult Glucagon-Like Peptide-1 Receptor Agonist Exposures Reported To United States Poison Centers, 2017–2024
Source: J Med Toxicol. 2026 Mar 5;22(2):263–74. doi: 10.1007/s13181-026-01128-6 (PMC13076819; doi:10.1007/s13181-026-01128-6)
Supplement: Supplementary file 1 — Supplementary Material 1 (DOCX 46.0 KB) [file 13181_2026_1128_MOESM1_ESM.docx]

**Appendix 1. Rate of More Serious Medical Outcomes Associated with GLP-1 Exposures Reported to United States Poison Centers by Sex, NPDS 2017-2024**

**Appendix 2. Rate of More Serious Medical Outcomes Associated with GLP-1 Exposures Reported to United States Poison Centers by Age Group, NPDS 2017-2024**

**Appendix 3. Rate of More Serious Medical Outcomes Associated with GLP-1 Exposures Reported to United States Poison Centers by Product Category, NPDS 2017-2024**

**Appendix 4. Rate of Medical Admissions Associated with GLP-1 Exposures Reported to United States Poison Centers by Sex, NPDS 2017-2024**

**Appendix 5. Rate of Medical Admissions Associated with GLP-1 Exposures Reported to United States Poison Centers by Age Group, NPDS 2017-2024**

**Appendix 6. Rate of Medical Admissions Associated with GLP-1 Exposures Reported to United States Poison Centers by Product Category, NPDS 2017-2024**
